# Newcastle disease virus induces testicular damage and disrupts steroidogenesis in specific pathogen free roosters

**DOI:** 10.1186/s13567-020-00801-0

**Published:** 2020-06-29

**Authors:** Zaib Ur Rehman, Shanhui Ren, Bin Yang, Xiaofeng Yang, Salman Latif Butt, Alia Afzal, Muhammad Irfan Malik, Yingjie Sun, Shengqing Yu, Chunchun Meng, Chan Ding

**Affiliations:** 1 Shanghai Veterinary Research Institute (SHVRI), Chinese Academy of Agricultural Sciences (CAAS), Shanghai, 200241 China; 2 Department of Poultry Science, Faculty of Veterinary and Animal Sciences, PMAS Arid Agriculture University, 46300 Rawalpindi, Pakistan; 3 Department of Pathology, College of Veterinary Medicine, University of Georgia, Athens, GA 30602 USA; 4 Institute of Statistics, Faculty of Economics and Management, Leibniz University Hannover, 30167 Hannover, Germany; 5 Jiangsu Co-innovation Center for Prevention and Control of Important Animal Infectious Disease and Zoonoses, Yangzhou University, Yangzhou, 225009 China

## Abstract

Newcastle disease (ND), which is caused by Newcastle disease virus (NDV), can cause heavy economic losses to the poultry industry worldwide. It is characterised by extensive pathologies of the digestive, respiratory, and nervous systems and can cause severe damage to the reproductive system of egg-laying hens. However, it is unknown whether NDV replicates in the male reproductive system of chickens and induces any pathologies. In this study, we selected a representative strain (i.e. ZJ1) of the most common genotype (i.e. VII) of NDV to investigate whether NDV can induce histological, hormonal, and inflammatory responses in the testes of specific pathogen free (SPF) roosters. NDV infection increased the expression of toll like receptor TLR3, TLR7, MDA5, IFN-α, IFN-β, IFN-γ, IL-8, and CXCLi1 in the testes of NDV-infected roosters at 5 days post-infection (dpi). Severe histological changes, including decrease in the number of Sertoli cells and individualized, shrunken spermatogonia with pyknotic nuclei, were observed at 3 dpi. At 5 dpi, the spermatogenic columns were disorganized, and there were fewer cells, which were replaced by necrotic cells, lipid vacuoles, and proteinaceous homogenous material. A significant decrease in the plasma concentrations of testosterone and luteinizing hormone (LH) and the mRNA expression of their receptors in the testes, steroidogenic acute regulatory protein, cytochrome P450 side-chain cleavage enzyme, and 3β-hydroxysteroid dehydrogenase in the NDV-infected group was observed relative to those in the control group (*P* < 0.05). Collectively, these results indicate that NDV infection induces a severe inflammatory response and histological changes, which decrease the steroidogenesis.

## Introduction

Newcastle disease (ND) is caused by *Avian orthoavulavirus 1* (AOAV 1) (formerly designated as *Avian avulavirus 1* (AAvV-1)), commonly known as *Avian paramyxovirus 1* (APMV-1), or Newcastle disease virus (NDV), and is a leading cause of economic losses to the poultry industry worldwide [1, 2]. APMV-1 belongs to the genus Avulavirus in the family Paramyxoviridae and order Mononegavirales, which encompasses a diverse group of non-segmented, single‐stranded, and negative‐sense RNA viruses [1, 3]. The 15 kb genome of NDV encodes six proteins, including the nucleocapsid, phosphoprotein, matrix (M), fusion, haemagglutinin-neuraminidase, and large polymerase, and uses host cellular machinery for translation after invasion.

NDV can infect almost every species of bird, and the virulent strains of NDV cause one of the most serious infectious diseases of commercial poultry [4]. Based on the clinical manifestations, NDV is divided into four pathotypes, which are listed as follows in increasing order of virulence: asymptomatic enteric, lentogenic, mesogenic, and velogenic [5, 6]. Based on tissue tropism, velogenic strains of NDV can be further divided into viscerotropic and neurotropic strains. NDV replicates in almost every organ and mainly affects the digestive, respiratory, and nervous systems and causes complex pathologies in these organs, which decrease growth and egg production.

In commercial poultry farming, broiler and layer breeders are reared to produce fertile eggs to obtain the broiler and layer chicks, which are eventually grown to meet the requirement of high-quality animal protein for human consumption. Many factors affect the fertility of roosters and hens. In roosters, semen production and quality is affected by infectious and non-infectious factors [7]. Infectious factors, such as avian leukosis virus [8], Marek’s disease virus [9], and NDV [10], affect reproductive performance. Although, NDV replicates in the ovary and oviduct [11], causing severe inflammation and apoptosis, and result into decreased egg production [12–14] and fertility rates [15], the pathologic effects of NDV on the male reproductive system are yet to be characterized.

Spermatogenesis (sperm production) is a complex, hormone-controlled process, and sperm cells are produced from the spermatogonial stem cells within the seminiferous epithelium. Spermatogenesis is meticulously managed by testosterone, follicle stimulating hormone (FSH), gonadotropin-releasing hormone (GnRH), luteinizing hormone (LH), germ cells, neurons within the central nervous system, and their interaction with Sertoli cells [16] and is ultimately controlled by the hypothalamus-pituitary–gonadal axis [17].

The innate immune response is the first line of defence against invading viruses and stimulates a specific mucosal and humoral immune response [18]. In NDV infection, after the detection of viruses by pattern recognition receptors (PRRs), complex host–pathogen interaction pathways direct an intense inflammatory response to inhibit viral replication [14, 19] and elevate plasma glucocorticoids [20]. These pro-inflammatory cytokines and their crosstalk with hormones shape the immune system to control the potential harmful effects and the return of homeostasis after the clearance of a pathogen [21, 22]. Similarly, glucocorticoids affect the hypothalamic-pituitary–gonadal axis to control the hypothalamus to synthesise and release gonadotropin releasing hormone and the pituitary gland to prevent the synthesis and release of LH, and FSH [23].

In poultry, studies of NDV pathogenesis have mainly focused on the intestines, lungs, trachea, brain, spleen and feathers [14, 24–29]. Studies on the effects of NDV infection on histological lesions, innate immune responses, and steroidogenesis in the testes are rare. Therefore, we harnessed the NDV infection in white leghorn rooster testes in this study.

## Materials and methods

### Virus and reagents

A wild-type velogenic NDV isolate, ZJ1, was originally isolated from geese in 2000 (Goose/China/ZJ1/2000; GB AF431744.3) and was generously provided by Professor Xiufan Liu from Yangzhou University (Yangzhou, China). The pathogenicity indices including the mean death time (MDT), intracerebral pathogenicity index (ICPI), and intravenous pathogenicity index (IVPI) of ZJ1 were 51.6, 1.89, and 2.7, respectively. ZJ1 belongs to sub-genotype VIId of genotype VII, and has been responsible for the recent disease outbreaks in Asian countries [30]. The virus stock was prepared by growing the virus in 10-day-old SPF embryonating chicken eggs and was subsequently stored at − 80 °C until further use.

### Animals, ethics statement and treatments

All the animal experimental procedures were performed in strict accordance with the recommendations in the Guide for the Care and Use of Laboratory Animals of the Shanghai Veterinary Research Institute (SHVRI, Shanghai, China) of the Chinese Academy of Agricultural Sciences (CAAS, Beijing, China). All the protocols applied in this study were approved by the Institutional Animal Care and Use Committee of SHVRI (Permission number: SHVI-RO-2018030178), CAAS. All efforts were made to minimize the suffering of birds.

SPF white leghorn roosters were purchased from Zhejiang Lihua Agricultural Technology Co. Ltd (China) and reared up to the age of 25 weeks before the collection of blood samples or the challenge studies. Birds were housed in positive pressure isolators, and a photoperiod of 16L:8D was set. All the birds were provided with ad libitum access to feed and water throughout the experiment. At the age of 25 weeks, roosters were randomly divided into two groups of 20 chickens each. The birds in group 1 were mock infected with PBS and served as negative controls, whereas the birds in group 2 (NDV-challenged) were infected with 0.1 mL of a ZJ1 suspension containing a 10^5.5^ 50% embryo infectious dose via the right eye and choanal slit instillation.

Four birds per treatment were selected for daily blood and tissue sample collection. Blood samples were collected from the wing vein at 1, 3, and 5 days post-infection (dpi) in EDTA coated tubes and immediately transferred to the laboratory, maintaining the cold chain. To obtain the plasma, blood samples were centrifuged at 2000 × *g* for 10 min at 4 °C and stored at − 80 °C until analysis. A total of four chickens per experimental treatment were sacrificed every day for the collection of testes at 1, 3, and 5 dpi. One part of every tissue sample was rinsed with PBS and put in microtubes (already marked and weighed), immediately frozen in liquid nitrogen, and subsequently stored at − 80 °C until further use. Another part of each testis was fixed in 10% neutral-buffered formalin tubes for histological studies.

### Detection of viral loads in the testis

The NDV ZJ1 strain was grown in 10-day-old SPF embryonated eggs and allantoic fluid was collected after 60 h of infection. Viral RNA was extracted from the allantoic fluid using TRIzol reagent (Invitrogen, Carlsbad, CA, USA) as per manufacturer’s instructions. A 1095 bp fragment of the ZJ1 M gene was amplified, and electrophoresis was performed to know the correct size of the product. The PCR product was purified with the HiPure Gel Pure DNA Mini Kit (AnGen Biotech, Guangzhou, China) as per manufacturer’s instructions and cloned into a plasmid vector to construct a standard curve. Approximately, a total of 1 μg RNA extracted from the testis samples was reverse transcribed to cDNA with HiScript II (Catalogue # R233; Vazyme Biotech Co., Ltd., China), and quantitative PCR for NDV was performed with SYBR Premix (Dongsheng, Biotech, China). Virus copy numbers were calculated using the standard curve.

### Histology of testis

Tissue samples collected at 3 and 5 dpi were fixed in 10% neutral buffered formalin, processed by a standard histological procedure. Sections with a thickness of 5 µm were cut and stained with haematoxylin and eosin [31]. The slides were scanned and digitalized with Panoramic SCAN (3DHISTECH Ltd., Hungary), and histopathological lesions were evaluated with CaseViewer 2.2 (3DHISTECH Ltd.).

### Hormone analysis

Specific radioimmunoassay kits were used to determine plasma hormone concentrations. All samples were analysed in 1 assay to avoid inter-assay variations. Pre-experimental evaluations were conducted for all hormones to measure the optimum dilution of plasma to determine their concentration. The concentrations of testosterone (CSB-E12797C) were determined using commercially available chicken specific kits (CUSABIO, Wuhan, China) following the manufacturer protocols. The plasma LH levels of roosters were determined using an ELISA kit (MBS008505; MyBioSource, San Diego, CA, USA) according to the manufacturer’s protocol. The minimum detectable limit of LH was less than 1.0 mIU/mL. The absorbance was determined at 450 nm using an Epoch microplate spectrophotometer (BioTek Instruments, Inc., Winooski, VT, USA). The concentrations of testosterone and LH were calculated by the equation developed from the values of standards provided with the respective kits.

### RNA isolation, reverse transcription, and relative gene expression/quantitative real time PCR

Quantitative RT-PCR (qRT-PCR) was performed to determine the mRNA expression levels of selected genes. Chicken GAPDH gene was used as endogenous control. The primers used in the present study are described in Table 1.

**Table 1:** **Primer sequences used for quantitative PCR**

Gene type	Forward primer (5′–3′)	Reverse primer (5′–3′)	Amplicon size (bp)	Gen Bank accession number
ZJ1 M gene	ATGGACTCATCCAGGACAATCGGGCT	TTATTTCCTGAAAGGATTGTATTTAGCAATGG	1095	AF431744.3
ZJ1 M gene	TACTTTGATTCTGCCCTCCCTT	TAAGCAGAGCATTGCGGAAGA	255	AF431744.3
CXCLi2/IL-8	CATCATGAAGCATTCCATCT	CTTCCAAGGGATCTTCATTT	205	NM_205498.1
CXCLi1	CCGATGCCAGTGCATAGAG	CCTTGTCCAGAATTGCCTTG	191	NM_205018.1
TLR3	ACAATGGCAGATTGTAGTCACCT	GCACAATCCTGGTTTCAGTTTAG	189	XM_025149682.1
TLR7	TGTGATGTGGAAGCCTTTGA	ATTATCTTTGGGCCCCAGTC	219	XM_015273651.2
MDA-5	GGACGACCACGATCTCTGTGT	CACCTGTCTGGTCTGCATGTTATC	79	NM_001193638.1
IFN-α	GGAGTTTTGAGGAGGGTGGG	CGCGTCTTCCTTCCTCCTTT	175	XM_004937092.3
IFN-β	AACACTGGATTGACCGCACA	GTCCCAGGTACAAGCACTGT	200	NM_001024836.1
IFN-γ	TGAGCCAGATTGTTTCGATG	CTTGGCCAGGTCCATGATA	152	NM_205149.1
Androgen receptor (AR)	AGTGCCAGCCCATCTTTCTC	CCTTTGCCCACTTGACGAC	159	NM_001040090.1
LH receptor (LHR)	ACTCCTGCGCAAACCCATTC	CTCGGCTCTTACAGCAACCT	99	NM_204936.1
StAR	TTCAGCGAGATGGAGATGTCC	GGAACACCTTACCCACGTCC	160	NM_204686.2
3b-hydroxysteroid dehydrogenase (3βHSD)	GGGCAAGACTGAGGTGAAAATC	TGTGTGGATGACGAGCGAG	94	XM_015294370.2
Gallus gallus hydroxysteroid 17-beta dehydrogenase 4 (17βHSD4)	CGCTGGAGGAGGTTTGGG	TGGGTACTGCTTTCCCTCCA	167	NM_204943.1
Cholesterol side-chain cleavage enzyme, P450scc	GTTGGGTGTCTACGAGAGCG	TTGCGGTAGTCACGGTATGC	126	NM_001001756
β-actin	GGTCATCACCATTGGCAATG	CCCAAGAAAGATGGCTGGAA	66	L08165
GAPDH	CCATCACAGCCACACAGAAGAC	TGGACGCTGGGATGATGTT	93	NM_204305

Total RNA was extracted from the frozen tissue samples using TRIzol reagent (Invitrogen, Carlsbad, CA, USA) following the manufacturer’s protocol. The quality and purity of the extracted RNA were examined spectrophotometrically (NanoDrop spectrophotometer Thermo Fisher Scientific, Waltham, MA, USA) by determining the ratio of absorbance at 260 to 280 nm. Samples with a 260/280 ratio of 1.8–2.0 were selected for reverse transcription. Purified RNA was dissolved in RNase-free water and immediately used as templates in reverse transcription. Briefly, 1 μg of total RNA was mixed with 2 μL gDNA wiper Mix and 0.5 μL of random hexamers, and the total reaction volume was brought to 8 μL by adding RNase free ddH_2_O. The mixture was heated at 42 °C for 2 min and then transcribed to cDNA with HiScript II (Catalogue # R233; Vazyme Biotech Co., Ltd. China) at 55 °C for 15 min and 85 °C for 2 min. The prepared cDNA samples were stored at − 25 °C until analysis.

Real-time qRT-PCR was performed using SYBR Premix (Dongsheng Biotech, Guangzhou, China) and a CFX96 Touch Real-Time PCR Detection System (BioRad, USA). A final volume of 20 µL was used for qRT-PCR. The PCR cycles were as follows: 94 °C for 3 min, followed by 40 cycles of 95 °C for 15 s, 60 °C for 15 s, and 72 °C for 20 s. The melting temperature of the final double-strand DNA product was determined by intercalated SYBR Green at the end of the reaction. PCR efficiencies were above 1.85, and amplifications generated single expected amplicons with single, sharp fusion curves. All experiments were performed in triplicate. The changes in mRNA levels were presented as fold expression and calculated using the 2^−ΔΔCT^ method [32].

### Statistical analysis

The data was analysed by a two-way ANOVA with challenge and time points as the main effects [33], and Graph Pad Prism 6.0 software (GraphPad Software, Inc., CA, USA) was used to generate the graphs. The graphical results were expressed as mean ± standard deviation. Results with *P* < 0.05 were considered statistically significant. When a significant main effect was observed, the Tukey test was used to compare the differences among groups.

## Results

### Histopathological findings

**Figure 1:**
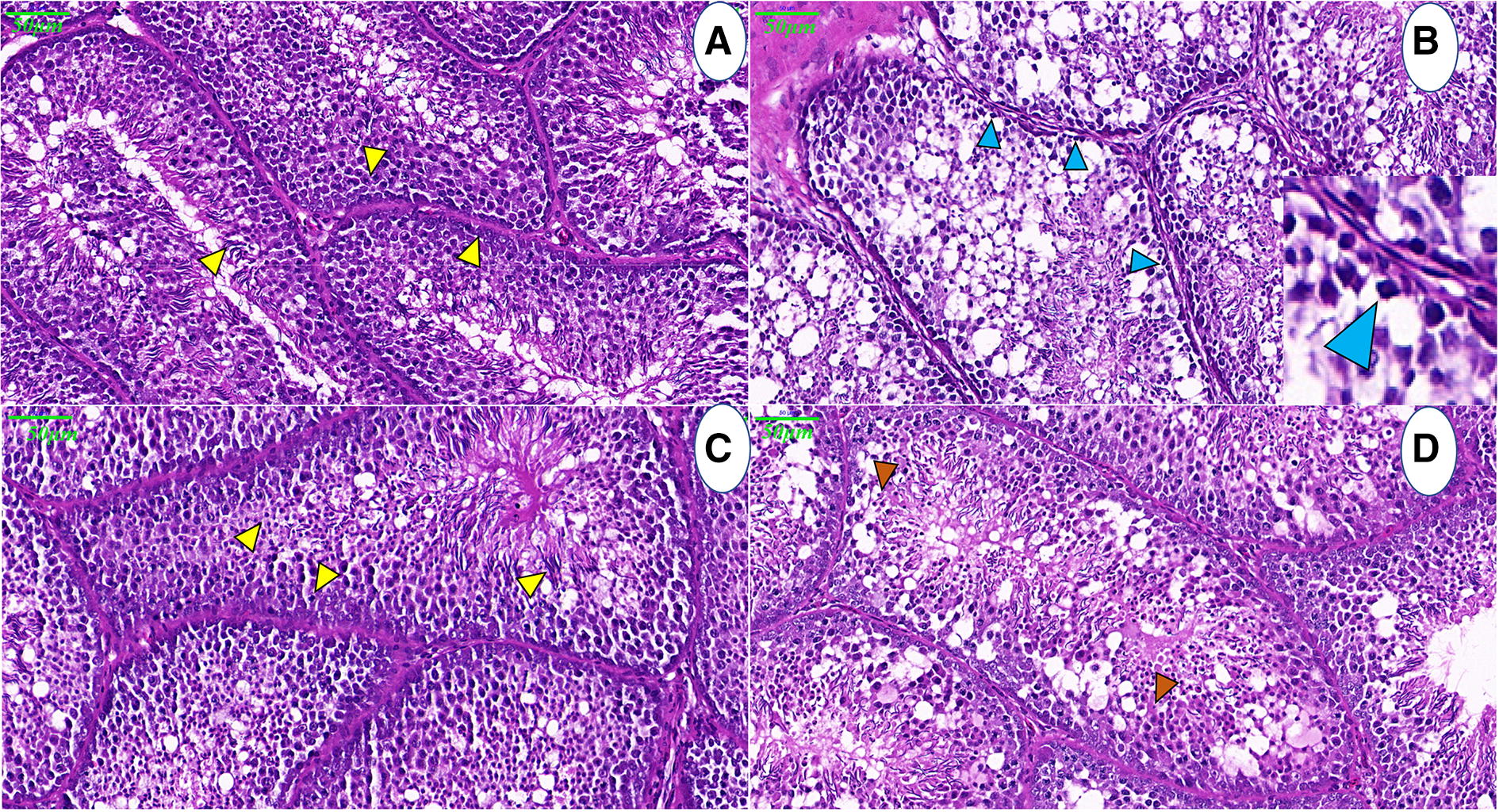
**Photomicrograph of NDV induced histopathological changes in the testis of control and NDV infected white leghorn roosters.** Normal progression of spermatogenesis (yellow arrows pointing various stages), with the formation of mature spermatids, few are attached with Sertoli cells, and small numbers of mature spermatids were present in the lumen of seminiferous tubules of control birds, at 3 and 5 dpi (**A** and **C**). Panel **B** illustrates the shortening of the adluminal compartment of seminiferous epithelium, decrease in number of Sertoli cells, individualized, shrunken spermatogonia with pyknotic nuclei (blue arrow heads), at 3 dpi. The inset shows individualised spermatogonia. Histology of testis at 5 dpi, denotes multifocal, and segmented, spermatogenic columns, necrotic cells (brown arrow heads), lipid vacuoles and proteinaceous homogenous material. Sertoli cells were rare and small numbers of spermatids were present in the lumen (**D**).

The normal progression of spermatogenesis involves the formation of mature spermatids (yellow arrows pointing various stages) with few attached to Sertoli cells, and few mature spermatids were present in the lumen of seminiferous tubules of the control birds. These were admixed with scant proteinaceous matrix substance and few sloughed cells in the lumen in control roosters at 3 and 5 dpi (Figures 1A and C). The seminiferous tubules were separated by fine fibrovascular stroma.

At 3 dpi, the main microscopic lesions in roosters consisted of multifocal, individual spermatogonia at the basal layers of the seminiferous tubules, individualized and shrunken with pyknotic nuclei (blue arrow heads; Figure 1B). Numerous spermatogenic columns were disorganized and shortened with numerous necrotic cells filling the lumen admixed with scant spermatids. The number of Sertoli cells decreased, and there were fewer attached, late stage spermatids.

The histological study of rooster testes at 5 dpi (Figure 1D) indicated multifocal, disorganized, and segmented spermatogenic columns, and there were fewer cells, which were replaced by necrotic cells (brown arrow heads), lipid vacuoles, and proteinaceous homogenous material. Sertoli cells were rare, and few spermatids were present in the lumen. In most severely affected tubules, only a single layer of spermatogonia remained in the basal layer. The interstitium mildly expanded with oedema. Few multinucleated cells were observed in the lumen, and few of them were necrotic (Figure 1D).

### Quantification of viral RNA

**Figure 2:**
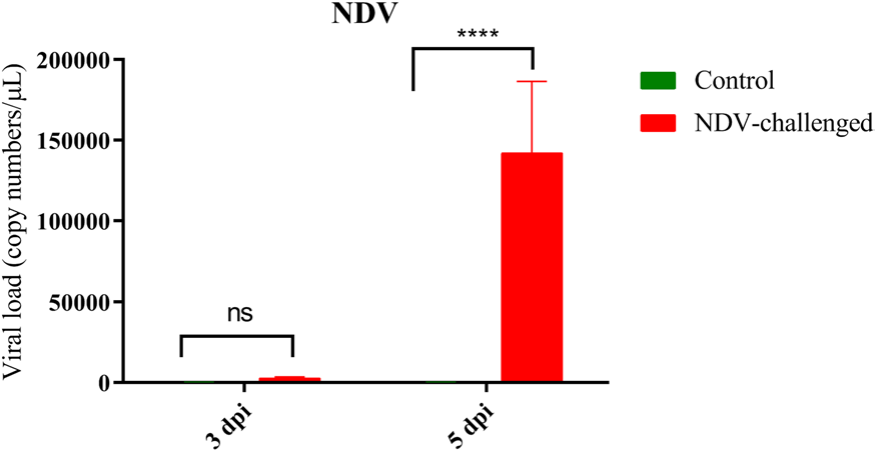
**Relative quantity of Viral RNA in the testis of roosters.** Graphs are denoted as mean ± standard deviation. The data were analysed by two-way ANOVA test, with challenge and time points as the main effects.

Virus titres were determined by isolation of RNA from the testicular tissue. Chicken embryos did not die after inoculation of tissue homogenates from the control birds. However, all the embryos died after inoculation of eggs with testes homogenates of NDV-infected birds at 5 dpi. Viral RNA expression was analysed by qRT-PCR. There was a significant increase in the level of NDV M gene expression at 5 dpi. There were approximately 15,000 copies at 5 dpi compared to the control group (Figure 2).

### Effect of NDV on the sex hormones and receptors

**Figure 3:**
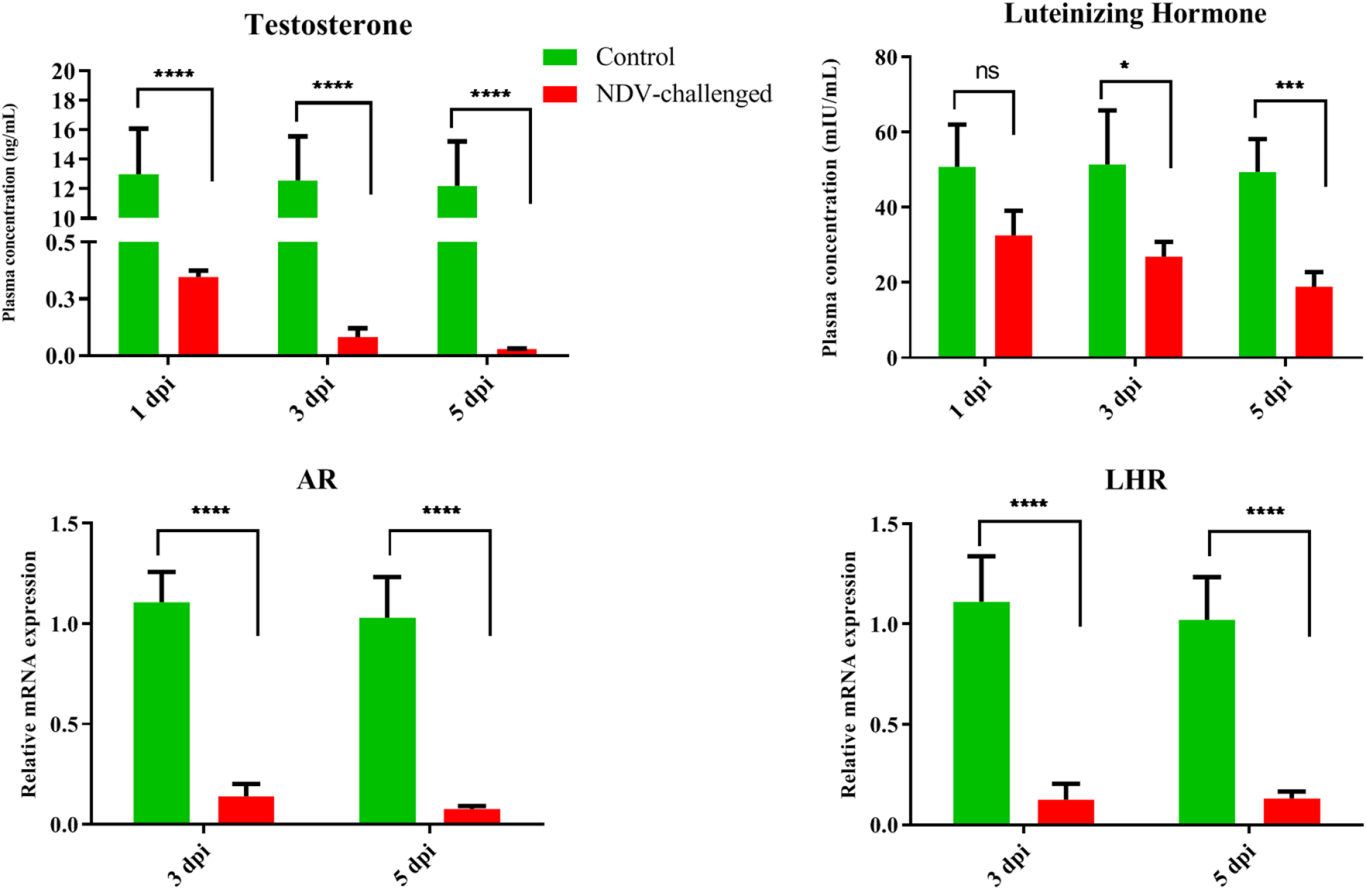
**Plasma concentrations of testosterone and luteinizing hormone and expression of their receptors in the control and NDV infected white leghorn roosters.** Plasma levels of steroids were determined by enzyme-linked immunosorbent assay and expression analysis were performed by qPCR. Data are presented as mean ± standard deviation. The data were analysed by two-way ANOVA test, with challenge and time points as the main effects.

The plasma concentration of testosterone significantly (*P* < 0.05) decreased in the NDV-infected roosters compared to the control group at 1, 3, and 5 dpi (Figure 3). There was a non-significant interaction between the infection and dpi for testosterone. A decrease in the plasma concentrations of LH was observed in the NDV-infected birds, but these levels were only statistically significant at 3 and 5 dpi (Figure 3). The plasma LH concentrations decreased in the NDV-infected birds compared to the control birds, particularly at 3 and 5 dpi. The interaction effects of dpi and NDV infection were non-significant.

The relative mRNA expression levels of the androgen receptor (AR) and LH receptor (LHR) in the testicular tissue are shown in Figure 3. The mRNA expression levels of AR were significantly (*P* < 0.05) decreased in the testicular tissue of the NDV-infected birds, but this decrease was more pronounced at 5 dpi. LHR expression in the control birds was significantly (*P* < 0.05) higher than that in the-NDV infected birds (Figure 3).

### Effect of NDV infection on mRNA expression of steroidogenesis related protein and enzymes

**Figure 4:**
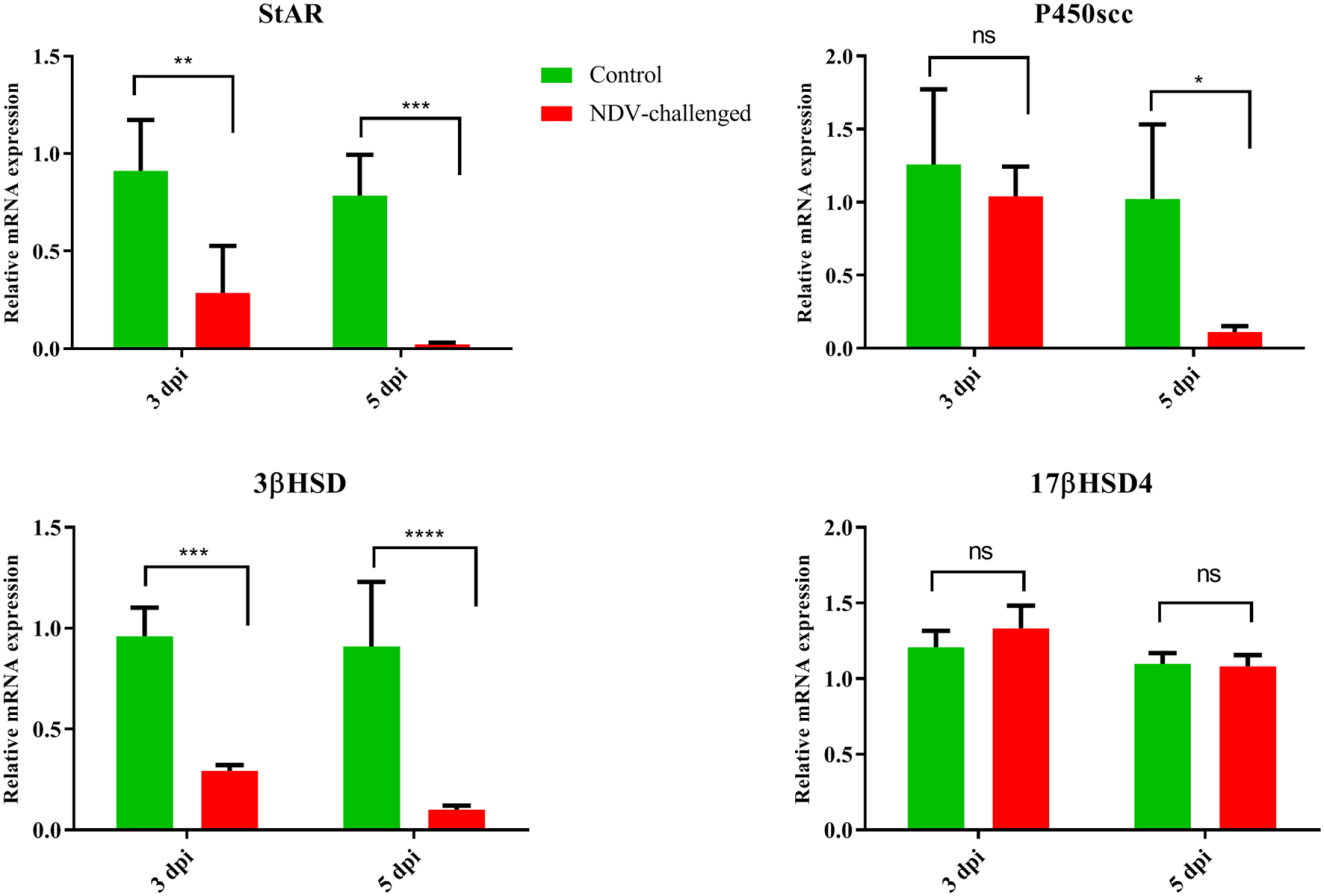
**The effects of NDV infection on the mRNA expression of steroidogenesis related key protein StAR and enzymes P450scc, 3βHSD, and 17βHSD4 in rooster testis.** Data are presented as mean ± standard deviation. The data were analysed by two-way ANOVA test, with challenge and time points as the main effects. StAR: Steroidogenic acute regulatory protein; P450scc: Cholesterol side-chain cleavage enzyme; 3βHSD: 3β-hydroxysteroid dehydrogenase.

To verify the results of the plasma hormones, expression analysis of P450scc, StAR, and 3βHSD was performed by qRT-PCR. NDV infection decreased the mRNA expression of P450scc in the NDV-infected group (*P* < 0.05) compared to the control group. Similarly, decreased expression of the 3βHSD was observed in infected birds at 3 and 5 dpi, as compared to non-infected birds (Figure 4). This decrease was more pronounced at 5 dpi than at 3 dpi. Experimental infection of roosters with NDV also decreased the expression (*P* < 0.05) of StAR in the testes compared to roosters in the control group. Although decreased expression was observed at both 3 and 5 dpi, it was lower at 5 dpi (Figure 4).

### NDV infection upregulated the mRNA levels of PRRs

**Figure 5:**
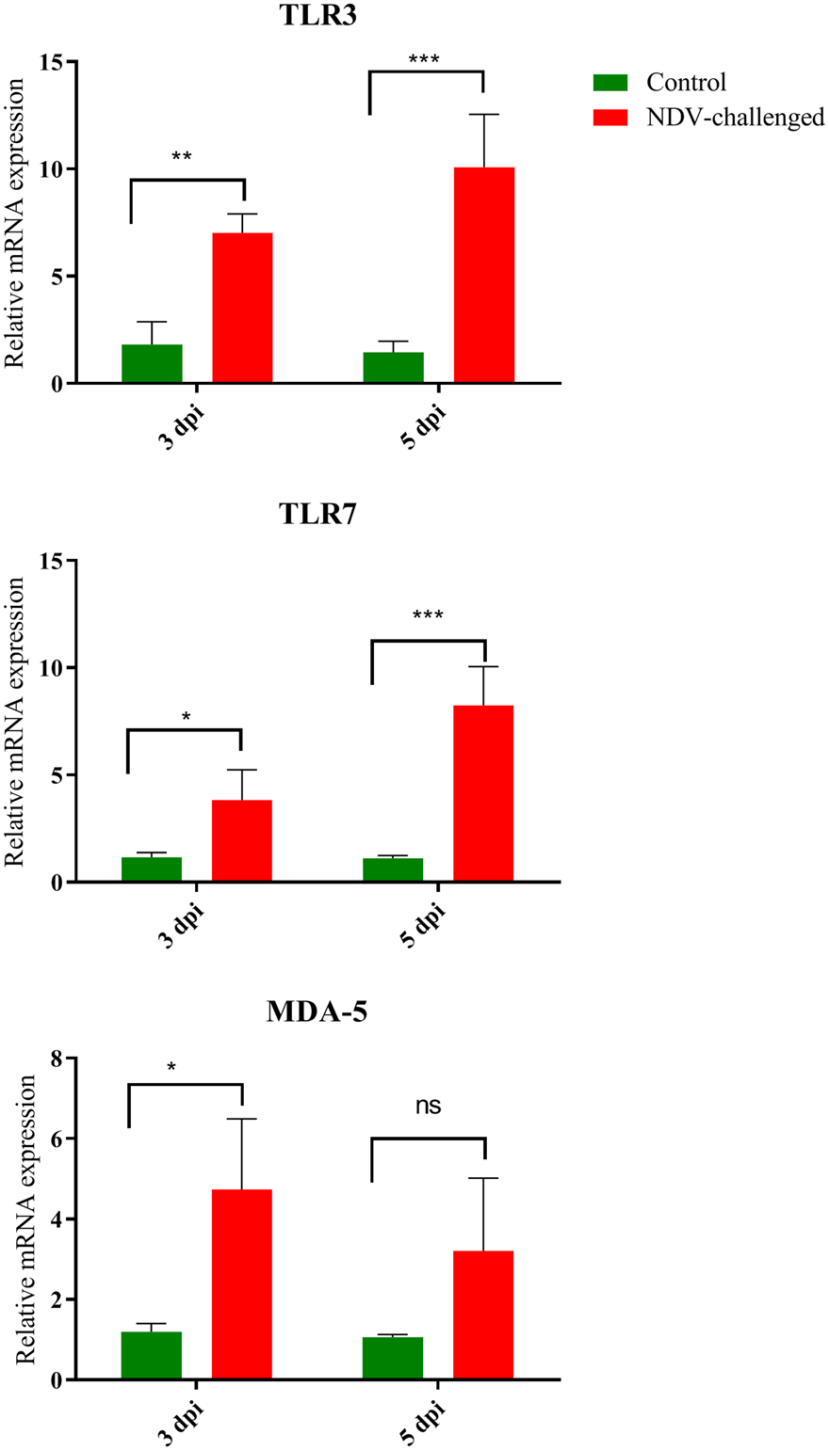
**Relative quantity of mRNA expression of Pattern recognition receptors in the testis of control and NDV infected birds.** Graphs are denoted as mean ± standard deviation. The data were analysed by two-way ANOVA test, with challenge and time points as the main effects.

We compared the mRNA expression levels of PRRs, including toll-like receptor TLR 3, TLR7, and melanoma differentiation-associated protein 5 (MDA5) in the testicular tissue of NDV-infected and control birds at 3 and 5 dpi. Compared to roosters in the control group, the expression levels of TLR3 and TLR7 in the testes of NDV-infected birds were significantly upregulated at 5 dpi, but these upregulations were only significant for TLR3 at 3 dpi (Figure 5). As shown in Figure 5, MDA5 was significantly upregulated in the NDV-infected group at 3 dpi compared to the control group. The upregulation of MDA5 in the NDV-infected group at 5 dpi was not significantly higher than that in the control.

### Differential expression of innate immune genes

**Figure 6:**
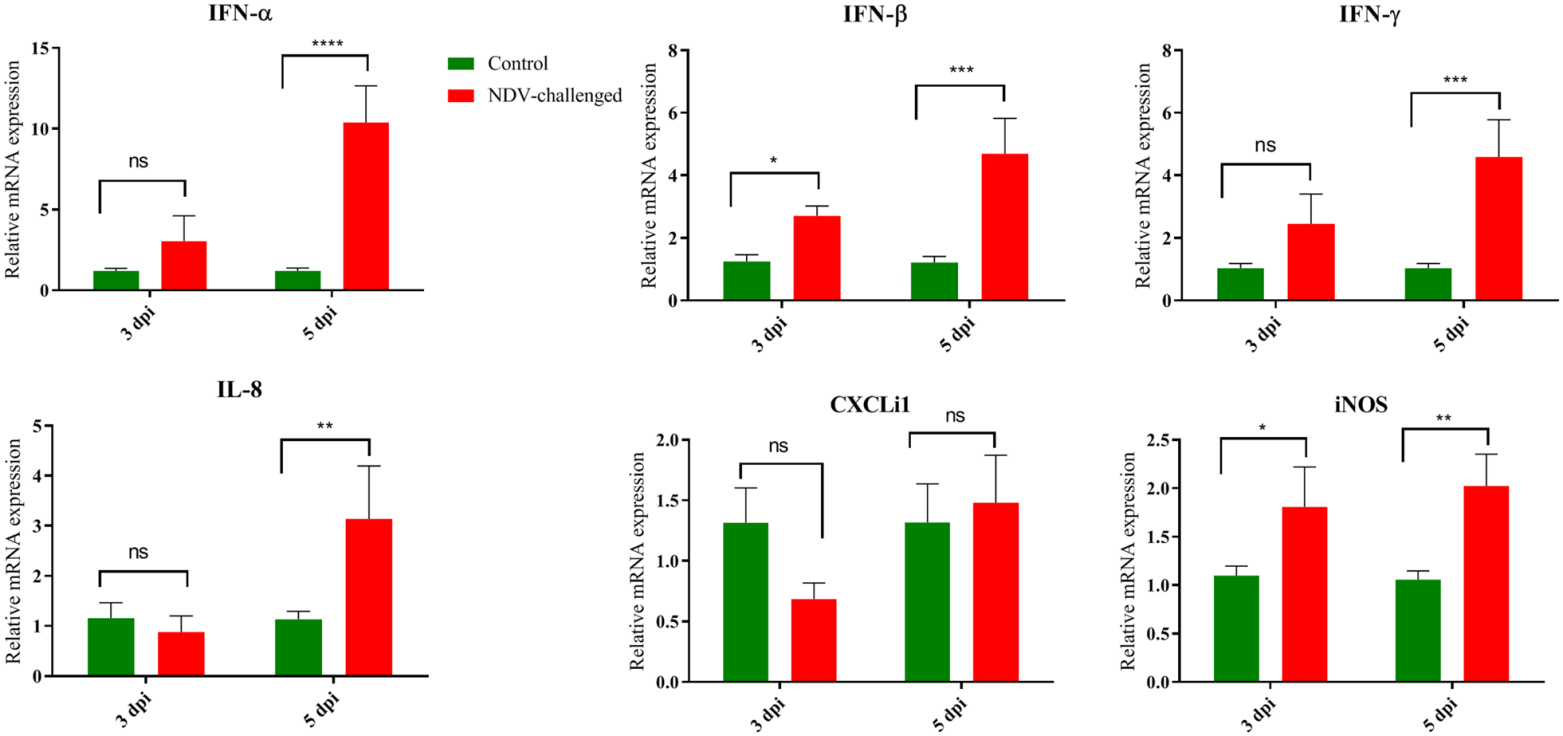
**Relative quantity of mRNA expression of innate immune genes in the testis of control and NDV infected birds.** Graphs are denoted as mean ± standard deviation. The data were analysed by two-way ANOVA test, with challenge and time points as the main effects.

Interferons (IFNs) are the most potent innate immune molecules to control and surpass viral replication and modulate innate immune responses to protect the host from viral pathogens. Therefore, we compared the expression of IFNs, including IFN-α, IFN-β, and IFN-γ, in the testes of roosters from the control and NDV-infected groups at 3 and 5 dpi (Figure 6). The expression of IFN-α, IFN-β, and IFN-γ was upregulated at 3 dpi, but these upregulations were only significant at 5 dpi (Figure 6). The expression levels of IL-8, CXCLi1, and iNOS were also examined by qRT-PCR. NDV infection significantly increased the expression of IL-8 at 5 dpi. NDV infection significantly (*P* < 0.05) increased the expression of iNOS in the infected roosters. The mRNA expression levels in the testes of NDV-infected birds peaked at 5 dpi. However, the expression of CXCLi1 decreased at 3 dpi in the testes of NDV-infected birds and increased at 5 dpi (Figure 6).

## Discussion

In the present study, we investigated the effects of NDV infection on the expression of PRRs, innate immune genes, enzymes involved in steroidogenesis, reproduction hormones levels, and histopathology of testes in SPF roosters. Many studies have attempted to explain the modulation of innate immune genes and histological structure in different organs and organ systems, including the digestive system, respiratory system, spleen, nervous system and female reproductive system [14, 24–28]. However, to the best of our knowledge, an attempt has never been made to study the fate of NDV infection in the testes of roosters.

Innate immune responses are the first line of defence of the host against invading pathogens and play a critical role in the determination of disease. Viruses are recognized by the PRRs, including TLRs, cytosolic retinoic acid inducible gene I (RIG-I)-like receptors, and MDA5 [34, 35]. In this study, increased expression of TLR3, TLR7, and MDA5 was noted in the testicular tissues of NDV-infected roosters. In NDV infection, upregulation of TLR3, TLR7, and MDA5 was noted in the DF-1 cells and different tissues [14, 36–39], which is in accordance with the results of the present study. Overexpression of TLR activates the MyD88-dependent pathway to produce cytokines, MHC molecules, and chemokines, leading to effective immune responses to clear invading pathogens [40, 41]. In this study, increased expression of CXCLi1 and IL-8 was noted in the testes of NDV-infected roosters. Upregulation of CXCLi1 and IL-8 might trigger immune cells at the infection site.

IFNs are part of the arsenal of the innate immune system against viruses [42]. Increased expression of IFNs has been noted in NDV infection [14, 36, 43, 44]. IFN production stimulates neighbouring cells to increase the expression of IFN-stimulated genes, leading to an antiviral state to inhibit virus replication [45]. Increased expression levels of IFN-α, IFN-β, and IFN-γ were noted in the testes of NDV-infected birds in the present study.

The ratio of roosters to laying hens is low, but roosters are equally important to produce fertile eggs and the subsequent performance of chicks. Different factors, such as nutrition [46–48], age [49, 50], hormones [51, 52], and infectious diseases [53–56], can affect the reproductive performance of roosters. Spermatogenesis is controlled by an array of pathways, especially by those including reproductive hormones. It takes place in the seminiferous epithelium and is controlled by the activity of Sertoli cells and their interaction with germ cells, testosterone, FSH, LH, oestradiol, and progesterone. In this study, decrease in the plasma levels of testosterone and LH in NDV-infected birds is indicative of disruptive effect of NDV on spermatogenesis. Decreased levels of testosterone have been shown to be associated with decreased fertility in roosters [52, 57].

In this study, examination of the viral load showed that NDV replicates in the testes of SPF roosters. Severe histological lesions were noted in the testes of NDV-infected roosters at 5 dpi than at 3 dpi, which may have been due to more viral copies. The optimum level of reproductive hormones ensures the spermatogenesis, however, disorganized spermatogenic columns and fewer cells with replacement by necrotic cells, lipid vacuoles, and proteinaceous homogenous material in the infected roosters are well correlated with the viral load and lowered level of reproductive hormones. The presence of rare Sertoli cells, few spermatids in the lumen, and severely affected tubules, only a single layer of spermatogonia remained in the basal layer could be explained with the fact that testosterone and FSH levels were lowered in the NDV infected birds. These microscopic lesions may have disturbed the plasma hormone levels, as the affected cells were associated with the production of testosterone.

Testosterone is produced from cholesterol through a series of reactions catalysed by different enzymes and proteins. The key enzymes and proteins involved in the synthesis of testosterone are P450scc, 3βHSD, and StAR [58]. In the Leydig cells, translocation of cholesterol from the outer to the inner mitochondrial membrane is regulated by StAR [59]. Then, conversion of cholesterol to pregnenolone is catalysed by P450scc [59]. StAR and P450scc are the rate limiting factors in pregnenolone synthesis. Then, pregnenolone, a precursor for the synthesis of diverse steroids, moves to the smooth endoplasmic reticulum and is converted to dehydroepiandrosterone and then androstenedione by 3βHSD. The final conversion of androstenedione to testosterone is catalysed by 17βHSD [60]. In the current study, NDV infection significantly downregulated the expression of StAR, P450scc, and 3βHSD. These results indicate that decreased mRNA expression of StAR, P450scc, and 3βHSD may have been the cause of decreased biosynthesis of hormones because the synthesis and levels of testosterone are closely associated with the expression of StAR, P450scc, and 3βHSD [58, 60]. Steroid synthesis is regulated by steroidogenic factor-1 [61].

LH stringently controls steroidogenesis. In the present study, NDV infection decreased expression of LHR and AR and the plasma levels of LH and testosterone. In addition to steroidogenesis, depletion of AR in murine Leydig cells inhibits spermatogenesis in the spermatid stage, decreases plasma testosterone levels, and reduces several key steroidogenic enzymes, including 17βHSD, 3βHSD, and P450scc [62]. Therefore, the decreased expression of steroidogenic enzymes in NDV-infected birds may have been due to decreased expression of LHR and AR.

In conclusion, our results demonstrate that NDV replicates in the testicular tissue, increases the expression of PPRs, upregulates the innate immune response, induces histological lesions, and inhibits steroidogenesis and spermatogenesis. These observations suggest that decreased plasma levels of testosterone and LH may be due to decreased expression of LHR and AR and steroidogenic enzymes in NDV-infected roosters. These findings also explain the possible reduction in the fertility and hatchability in NDV infected breeder flocks.

## Abbreviations

ND: Newcastle disease
NDV: Newcastle disease virus
TLR: Toll like receptor
MDA5: Melanoma differentiation-associated protein 5
IFN: Interferons
IL-8: Interleukin 8
LH: Luteinizing hormone
dpi: Day post-infection
StAR: Steroidogenic acute regulatory protein
P450scc: Cytochrome P450 side-chain cleavage enzyme
3βHSD: 3β-Hydroxysteroid dehydrogenase
APMV-1: Avian avulavirus 1
FSH: Follicle stimulating hormone (FSH)
SHVRI: Shanghai Veterinary Research Institute (Shanghai, China)
CAAS: Chinese Academy of Agricultural Sciences
CON: Control
AR: Androgen receptor
LHR: Luteinizing hormone receptor
RIG-I: Retinoic acid inducible gene I
